# Combination Treatments of Plasma Exchange and Umbilical Cord-Derived Mesenchymal Stem Cell Transplantation for Patients with Hepatitis B Virus-Related Acute-on-Chronic Liver Failure: A Clinical Trial in China

**DOI:** 10.1155/2019/4130757

**Published:** 2019-02-04

**Authors:** Wen-xiong Xu, Hong-liang He, Shun-wen Pan, Yuan-li Chen, Mei-ling Zhang, Shu Zhu, Zhi-liang Gao, Liang Peng, Jian-guo Li

**Affiliations:** ^1^Department of Infectious Diseases, Third Affiliated Hospital of Sun Yat-sen University, Guangzhou, 510630 Guangdong, China; ^2^Guangdong Key Laboratory of Liver Disease Research, Third Affiliated Hospital of Sun Yat-sen University, Guangzhou, 510630 Guangdong, China; ^3^Department of Infectious Diseases, First Affiliated Hospital of USTC, Division of Life Sciences and Medicine, University of Science and Technology of China, Hefei, 230001 Anhui, China; ^4^Department of Laboratory Medicine, Third Affiliated Hospital of Sun Yat-sen University, Guangzhou, 510630 Guangdong, China

## Abstract

**Background:**

Hepatitis B virus-related acute-on-chronic liver failure (HBV-ACLF) is a common type of liver failure with a high mortality. This study aimed at investigating the safety and efficacy of the combination treatment of plasma exchange (PE) and umbilical cord-derived mesenchymal stem cell (UC-MSCs) transplantation for HBV-ACLF patients.

**Methods:**

A total of 110 HBV-ACLF patients treated in our hospital from January 2012 to September 2017 were enrolled into this trial and divided into the control group (*n* = 30), UC-MSC group (*n* = 30), PE group (*n* = 30), and UC-MSC + PE group (*n* = 20) based on their treatments. The hepatic function, coagulation, and virological and immunological markers were assessed at baseline and 30, 60, 90, 180, and 360 days. The endpoint outcomes were death and unfavorable outcome (need for liver transplantation or death).

**Results:**

The UC-MSC + PE group had the lowest rates of death and unfavorable outcome at 30 days, 60 days, and 90 days posttreatment among the four groups, but the difference did not reach significances. The multivariate logistic regression analysis demonstrated that hemoglobin, prothrombin activity, and MELD (model for end-stage liver disease) score were the independent factors associated with the unfavorable outcome (all *P* < 0.05). The levels of total bilirubin, alanine aminotransferase, aspartate transaminase, and MELD score were significantly decreased during treatments (all *P* < 0.05).

**Conclusion:**

UC-MSCs combined with PE treatment had good safety but cannot significantly improve the short-term prognosis of HBV-ACLF patients with as compared with the single treatment. The long-term efficacy should be further evaluated. This trial is registered with registration no. NCT01724398.

## 1. Introduction

Acute-on-chronic liver failure (ACLF) is a syndrome characterized by acute decompensation of chronic liver disease and organ/system failure(s), resulting in a high short-term mortality rate [1]. In China, hepatitis B virus-related ACLF (HBV-ACLF) is the most common type of liver failure due to the high incidence of hepatitis B virus infection [2]. It has been shown that the patients with HBV-ACLF have a significantly higher short-term mortality as compared with those with non-HBV- ACLF [3]. The 90-day mortality of HBV-ACLF is reportedly up to 50% to 70% [3, 4].

Although liver transplantation (LT) is considered the only curative therapy for HBV-ACLF [5], however, it is limited by organ shortage for transplantation. In addition to AL, other treatment options for HBV-ACLF include nucleos(t)ide analogues [6], immunomodulatory therapy [7], artificial liver support systems (ALSs) [8], and stem cell therapy [9–11]. The various ALSs, such as conventional dialysis, charcoal hemoperfusion, high-volume plasma exchange (PE), bioartificial livers, extracorporeal liver assist device, and extracorporeal organ perfusion [12] are mainly used as a bridge to LT by eliminating the toxins in the blood of liver failure patients [13].

With the advance of cell therapy, mesenchymal stem cell (MSC) transplantation has been adopted for the treatment of HBV-ACLF. Accumulating evidence has demonstrated that transplantation of bone marrow-derived MSCs [9] or umbilical cord-derived MSCs (UC-MSCs) [11, 14] significantly improves the hepatic function and survival rate in patients with HBV-ACLF.

Although PE therapy and MSC transplantation have been shown to improve the hepatic function as well as the short-term and long-term prognoses of patients with HBV-ACLF, however, there remains a large proportion of patients poorly responsive to these therapies [11, 14–16]. Therefore, it was proposed that PE therapy prior to MSC transplantation might further improve the therapeutic efficacy. However, the studies on the therapeutic efficacy of the combination of PE therapy and MSC transplantation are still extremely rare. Therefore, this study aimed at investigating the safety and efficacy of the combination treatment of PE treatment and UC-MSC transplantation in patients with HBV-ACLF.

## 2. Methods

### 2.1. Patients

**Figure 1:**
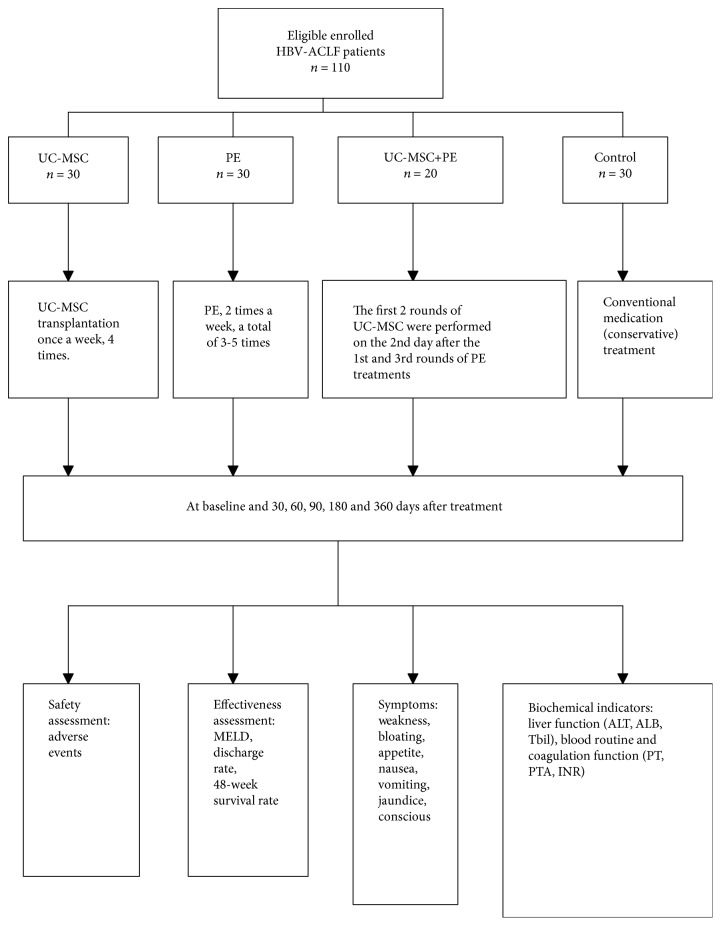
The consort diagram and flow chart of analysis.

This was a prospective clinical trial registered in ClinicalTrials.gov (registration no NCT01724398). A total of 110 HBV-ACLF patients treated in our hospital from January 2012 to September 2017 were enrolled. The inclusion criteria were as follows: (1) aged 19 to 64 years; (2) positive for hepatitis B virus surface antigen (HBsAg) for longer than six months; (3) coagulation disorders (international normalized ratio (INR) > 1.5 or prothrombin activity < 40%); and (4) severe jaundice (serum total bilirubin (TBIL) ≥ 10 × upper limit of normal (ULN)). The exclusion criteria were as follows: (1) combined with other hepatitis virus infection; (2) combined with autoimmune disease; (3) with a history of alcohol abuse or the use of hepatotoxic drugs in the past 6 months; (4) combined with heart and lung failure; (5) with a malignant tumor; (6) pregnant women or lactating women; and (7) imaging examinations indicating intrahepatic nodular space-occupying lesions. The consort diagram and flow chart of analysis is shown in Figure 1. This study was approved by the institutional review board (IRB) of the Third Affiliated Hospital of Sun Yat-sen University. All patients voluntarily signed an informed consent form approved by the IRB before participation.

### 2.2. Isolation and Culture of UC-MSCs

The isolation and culture of UC-MSCs were performed according to Good Manufacturing Practice (GMP) grade protocols in our GMP laboratory. After obtaining an informed consent from the donor parents, the umbilical cords were freshly harvested from full-term births at our hospital and placed in sterile containers. The arteries and veins were stripped, and the remaining tissue was immersed in PBS to wash out the remaining blood. The tissues were cut into small fragments and plated in a 50 mL tube and washed with PBS, followed by centrifuged at 200×*g* for 9 min. The resultant pellet was added with 10% volume of enzyme solution (0.1% type I collagenase and 0.1% hyaluronidase, Invitrogen, USA) and incubated on a shaker (220 rpm) at 37°C for 4 hours. After centrifugation at 200×*g* for 9 min, the resultant pellet was resuspended in growth media consisting of Dulbecco's modified Eagle's medium (DMEM; Invitrogen) with 10% fetal bovine serum (Invitrogen) and added to a T25 flask (Corning, USA). Cultures were then maintained at 37°C in a humidified atmosphere containing 5% carbon dioxide (*v*/*v*). The culture medium was changed at 5 days after plating and then changed every three days. The cells were subcultured at 80–90% confluence at a ratio of 1 : 3. UC-MSCs were characterized standard surface markers for MSC by flow cytometric analysis. The UC-MSCs should be positive (>90%) for CD90, CD105, and CD73 and negative (<2%) for CD45, CD34, CD19, CD14, and HLA-DR. Passage 3 UC-MSCs were used for transplantation.

### 2.3. Treatments

The 110 enrolled patients were randomly divided into the following four groups: Control group (*n* = 30): patients received conventional medication (conservative) treatment, such as polyene phosphatidylcholine, adenosyl methionine, dextromethorphan, compound glycyrrhizin tablets, ursodeoxycholic acid, glutathione, antibiotics, diuretics, aspartate ornithine, and lactulose. UC-MSC group (*n* = 30): in addition to conventional medication therapy, patients received allogeneic UC-MSC transplantation once a week for 4 weeks. PE group (*n* = 30): in addition to conventional medication therapy, patients were treated with plasma exchange (PE) in an artificial liver support system, 2 times a week, in a total of 3-5 times. PE + UC-MSC group (*n* = 20): in addition to conventional medication therapy, patients received a combination of UC-MSC transplantation and PE treatments. In the first two weeks, patients were given with PE treatment, 2 times a week, in a total of 3 times. At the second day of the first and third PE therapies, patients were treated with UC-MSC transplantation. In the third and fourth weeks, patients received UC-MSC transplantation once a week. No patients crossed over treatment allocations.

For UC-MSC transplantation, passage 4-6 UC-MSCs (10^5^ cells/kg) were resuspended in 100 mL of saline and transplanted by intravenous injection. The whole process of transplantation was about 30 minutes.

PE was performed by application of a double-filtration technique with a membrane plasmapheresis apparatus (Plasauto iQ21; Asahi Kasei Medical, Tokyo, Japan) with a plasma separator Plasmaflo OP-08 W (Asahi Kasei Medical), extracorporeal blood circuit PE-21C (Asahi Kasei Medical), and dual lumen dialysis catheter 11.5Fr (Lily Medical Technology Co., Ltd., Guangdong, China) according to the manufacturer's protocol. The total volume of plasma replacement was 2000 mL of fresh frozen plasma (FFP), with a blood flow of 100 mL/min and a plasma exchange rate of 25 mL/hour. The duration of each PE treatment was about 2 hours. Before and after PE treatment, patients routinely received 10 mL of 10% calcium gluconate as the antiallergic treatment.

### 2.4. Data Collection

Patients' demographic data and clinical characteristics were recorded. Clinical characteristics including regular blood testing, markers of hepatic function, and coagulation and virological and immunological markers were assessed at baseline and 30, 60, 90, 180, and 360 days. Complications (peritonitis, pneumonia, enteritis, gastrointestinal bleeding, hepatic encephalopathy, and hepatorenal syndrome) and treatment-related complications (fever, allergic reaction, and bleeding at the catheter insertion site) were also recorded. The MELD (model for end-stage liver disease) score was calculated using the following formula: MELD = 9.57 × log_e_(creatinine mg/dL) + 3.78 × log_e_(TBIL mg/dL) + 11.20 × log_e_(international normalized ratio (INR)) + 6.43 [17]. The endpoint outcome variables included death and unfavorable outcomes. Unfavorable outcome was defined as the need for liver transplantation or death. Outcome variables were recorded at baseline and 30, 60, 90, 180, and 360 days posttreatment.

### 2.5. Statistical Analysis

Continuous data were indicated with the mean ± SD while categorical data were reported with number and percentage (%). Two-way mixed-designed ANOVA was used to compare the means among the four groups and across time for repeated measurements. Fisher's LSD was used as a post hoc test. If normality was not assumed, nonparametric tests including the Kruskal-Wallis test, Friedman test, and Mann-Whitney test would be used. Categorical results were compared by a chi-square test or Fisher's exact test (if expected value < 5 was found). Associations between independent variables and outcome variable were analyzed using a univariate/multivariate generalized estimating equation (GEE) and logistic regression models. The first-order autoregressive working correlation matrix was adopted for the repeated measure data. Kaplan-Meier survival analysis was used to observe the univariate trend of group factor to outcomes. The statistical significance level for all the tests was set at a *P* value < 0.05. Statistical analyses were performed using IBM SPSS version 20 (SPSS Statistics V20, IBM Corporation, Somers, New York, USA).

## 3. Results

### 3.1. Patient's Characteristics at Baseline

A total of 110 patients with HBV-ACLF were included in this study, including 104 (94.55%) males and 6 (5.45%) females. The mean age was 42.14 ± 10.65. According to the treatments, patients were divided into four groups: control (*n* = 30), UC-MSC (*n* = 30), PE (*n* = 30), and UC-MSE + PE (*n* = 20). The patient's demographics, baseline clinical characteristics, and treatment-related complications (fever, allergic reaction, and bleeding at the catheter insertion site) are summarized in Table 1. Except for the incidence of gastrointestinal bleeding, neutrophil (N)% and albumin level, all the other characteristics did not significantly differ among the four groups (all *P* > 0.05), indicating that these four groups were mainly comparable.

### 3.2. Therapeutic Outcomes

**Figure 2:**
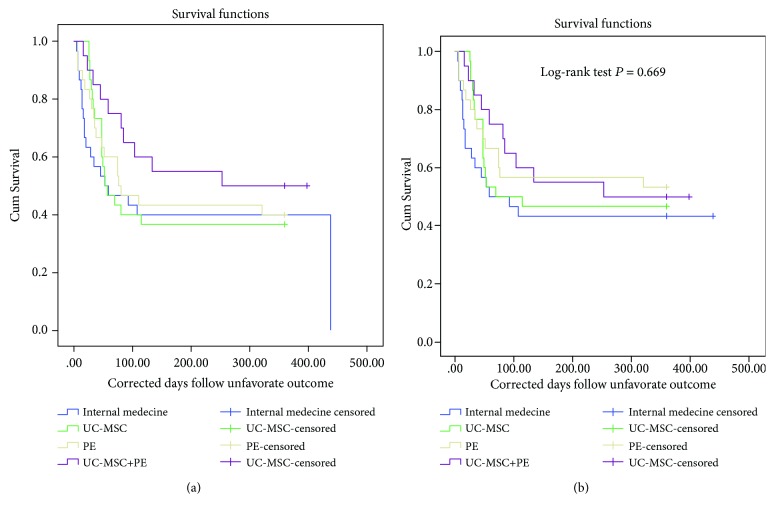
The Kaplan-Meier survival function among four treatment groups to unfavorable outcomes (a) and overall survival (b).

To evaluate the therapeutic efficacy, the unfavorable outcomes and survival rates were compared among the four groups. It was found that even though the PE + UC-MSC group had the lowest rates of unfavorable outcome (death and need for liver transplantation) at 30 days, 60 days, 90 days, 180 days, and 360 days posttreatment, however, there was no significance in both unfavorable outcome and survival rates among the four groups (all *P* > 0.05, Table 2). The Kaplan-Meier survival analysis also demonstrated that the unfavorable outcome (Figure 2(a)) and overall survival (Figure 2(b)) were not significantly different among the four groups (Log-rank test, both *P* > 0.05).

### 3.3. Independent Variables Associated with Unfavorable Outcomes

The independent variables associated with unfavorable outcomes were analyzed by logistic regression after modeling and adjusting covariates by GEE models. Variables with significance in the univariate analysis were included in the multivariate model. However, treatment variables (UC-MSC and PE) were included in the multivariate model because of the researcher's interest. The variables with significance in both univariate and multivariate analyses would be considered independent variables associated with unfavorable outcomes. As indicated in Table 3, complications, WBC, N%, hemoglobin, aspartate aminotransferase (AST), TBIL, creatinine, prothrombin activity, INR, and MELD score were significant variables in the univariate analysis (all *P* < 0.05). The variables TBIL, creatinine, and INR were not included into the multivariate model because the MELD score was calculated by these three variables and multicollinearity should be prevented. In the final multivariate model, UC-MSC and PE treatments were not significant in both univariate/multivariate results (all *P* > 0.05). The associated independent variables were hemoglobin (odds ratio (OR) = 0.98, 95% CI: 0.97-1.00, *P* = 0.035), prothrombin activity (OR = 0.93, 95% CI: 0.87-0.98, *P* = 0.01), and MELD score (OR = 1.12, 95% CI: 1.03-1.22, *P* = 0.008). These results suggested that a lower level of hemoglobin or prothrombin activity and a higher MELD score were associated with unfavorable outcomes.

### 3.4. Hepatic Function

**Figure 3:**
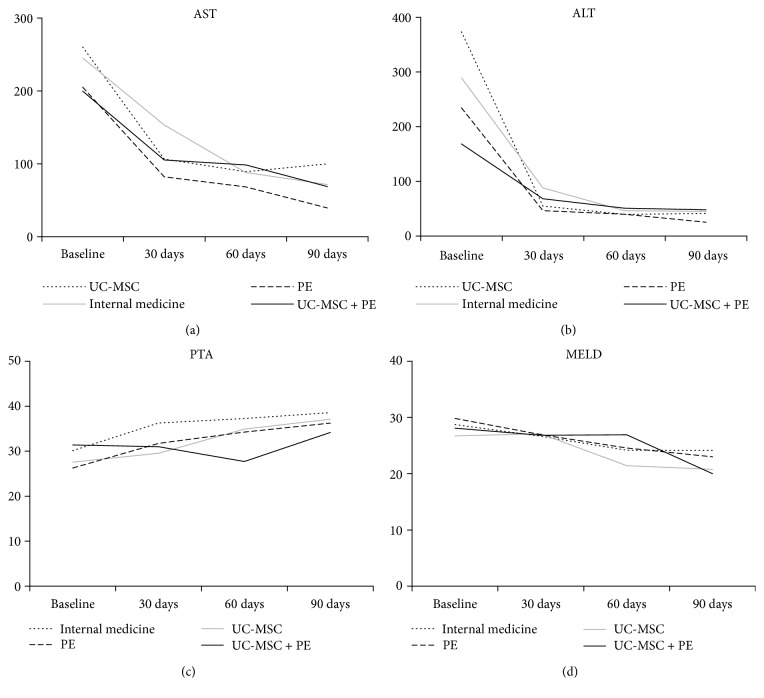
Biochemical markers of hepatic function and the severity of liver disease were shown from baseline to 90 days, including AST (a), ALT (b), prothrombin activity (c), and MELD score (d).

Next, we attempted to investigate if the treatments improve the hepatic function. Supplementary Tables S1 to S4 indicate the changes of the biochemical markers from baseline to month 3 among the four groups. In mix-designed two-way ANOVA analysis, the significant differences across time were found in WBC, N%, hemoglobin, platelet, AST, alanine transaminase (ALT), TBIL, and MELD score (all *P* < 0.05). However, group factor was not significant in all the 15 biochemical markers (all *P* > 0.05).  Figure 3 demonstrates the trends of AST (Figure 3(a)), ALT (Figure 3(b)), prothrombin activity (Figure 3(c)), and MELD score (Figure 3(d)). The obviously decreasing trends were found in AST, ALT, and MELD score.

**Figure 4:**
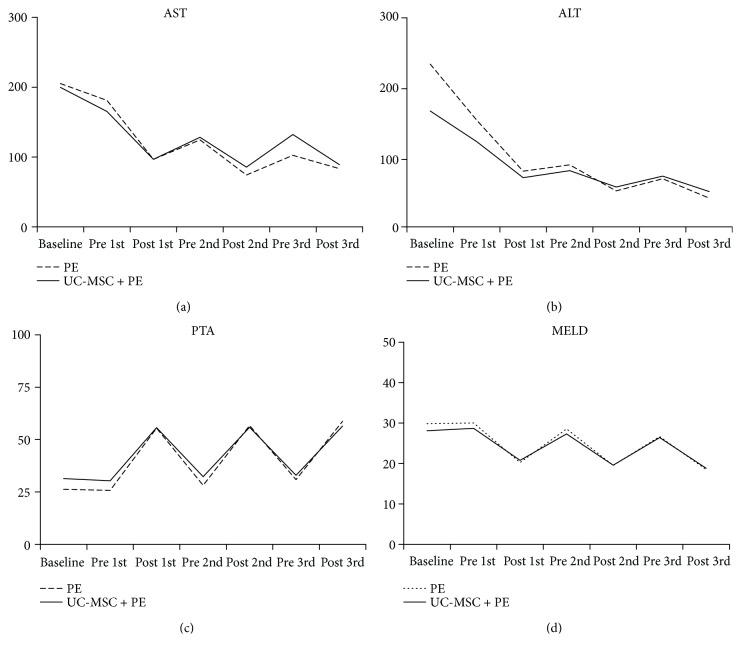
The changes of hepatic function markers were recorded before and, after 3 times of PE treatment, including AST (a), ALT (b), prothrombin activity (c), and MELD score (d).

The changes of biochemical markers before and after each PE treatment were further analyzed in the PE and PE + UC-MSC groups. As shown in Supplementary Tables S5 and S6, except for WBC, hemoglobin, albumin, and creatinine, all the other biochemical markers significantly differed before and after each PE treatment in both groups (all *P* < 0.05).  Figure 4 shows the trends of AST (Figure 4(a)), ALT (Figure 4(b)), prothrombin activity (Figure 4(c)), and MELD score (Figure 4(d)). Markedly higher levels before each PE treatment were found in AST, ALT, and MELD score while a lower level was found in prothrombin activity before each PE treatment.

## 4. Discussion

In this study, we investigated the safety and efficacy of the combination of PE treatment and UC-MSC transplantation in patients with HBV-ACLF. The results showed that the PE + UC-MSC group mainly had the lowest rates of death and unfavorable outcome at 30 days, 60 days, and 90 days posttreatment among the four groups, but the difference did not reach significances. Kaplan-Meier survival analysis also demonstrated the similar trends. The multivariate logistic regression analysis demonstrated that hemoglobin, prothrombin activity, and MELD score were the independent factors associated with the unfavorable outcome. The mix-designed two-way ANOVA analysis revealed that the levels of AST, ALT, TBIL, and MELD score were significantly decreased across time. In addition, after each PE treatment, the levels of AST, ALT, and MELD score were significantly reduced and the prothrombin activity was significantly elevated in the PE and PE + UC-MSC groups as compared with those before PE treatment. Taken together, our results suggested that UC-MSC treatment combined with PE treatment has good safety for patients with HBV-ACLF; however, it cannot significantly improve the short-term prognosis as compared with the single treatment.

Our previous trial found that transplantation of bone marrow-derived MSCs weekly for 4 weeks at the dose of 10^5^-10^6^ cells/kg for HBV ACLF significantly increases the 24-week survival rate by improving liver function and decreasing the incidence of severe infections [9]. Therefore, in this trial, we used the dose of 10^5^ cells/kg for UC-MSC transplantation. The studies on the therapeutic efficacy of the combination of PE therapy and MSC transplantation for HBV-ACLF are rare. Recently, Li et al. have conducted a prospective study to investigate the efficacy of UC-MSC transplantation combined with PE therapy for the patients with HBV-ACLF [11]. Their results showed that the PE + UC-MSC group (*n* = 11) has a significantly higher cumulative survival rate at 3 (54.5% vs. 29.4%) and 24 months (54.5% vs. 26.5%) as compared with the PE group (*n* = 34) [11]. In this study, even though the PE + UC-MSC group had the lowest incidence of unfavorable outcome at 30, 60, 90, 180, and 360 days posttreatment and the highest survival rate among the four groups at 30 days (survival rate = 90%), 60 days (survival rate = 75%), and 90 days (survival rate = 65%) posttreatment, nevertheless, there were no significances in both unfavorable outcome and survival rates among the four groups. This observation is in disagreement with Li et al.'s study [11]. The 90-day survival rate of the PE + UC-MSC group in our study was higher than the 3-month survival rate of those in Li et al.'s study (65% vs. 53.33%) [11]. However, the 90-day survival rate of the PE group in our study is higher than that in Li et al.'s study (56.67% vs. 29.4%), which should contribute to the insignificant result of the 90-day survival rate between the PE + UC-MSC group and the PE group in our study. In addition, we only followed up the patients for 360 days until now. A longer follow-up duration and a large sample size are necessary to comprehensively evaluate the effect of combination therapy.

In this study, a decreasing trend in the levels of AST, ALT, TBIL, and MELD score could be observed during the treatment course (from baseline, 30 days, 60 days, and 90 days) in the PE + UC-MSC group. In addition, after each PE treatment, the levels of AST, ALT, and MELD score were significantly reduced and the prothrombin activity level was significantly elevated. These results suggested that combination of PE therapy and MSC transplantation can improve the hepatic function of patients with HBV-ACLF, which is in line with Li et al.'s study [11].

The safety of treatment was evaluated by the treatment-related complications. After UC-MSC transplantation, 11 patients in the UC-MSC group and 6 patients in the PE + UC-MSC group had 16 and 7 fever episodes, respectively. All patients with fever returned to normal body temperature within 24 hours without any treatment. After PE therapy, 8 patients in the PE group and 6 in the PE + UC-MSC group experienced 9 and 6 allergic episodes due to a large amount of plasma exchange, respectively. The allergic reaction was resolved within 2 hours after treating with dexamethasone. Meanwhile, 4 cases in the PE group and 1 in the PE + UC-MSC group had bleeding at the catheter insertion site, which was resolved by compression bandages within 1 hour. Overall, these treatment-related complications were treated appropriately and cured within a short period and did not impact the patient's life. These observations suggest that UC-MSC transplantation combined with PE therapy has good safety, which is consistent with Li et al.'s study [11].

Several limitations of this study should be pointed out. First, the patients in this study were followed up for only 360 days until now. In addition, the sample size was small. Therefore, a well-designed study with a large sample size and long-term follow-up is necessary to further evaluate the efficacy of the therapeutic efficacy of the combination of PE therapy and MSC transplantation in patients with HBV-ACLF. All these limitations should be addressed in the following study.

## 5. Conclusions

In summary, our results showed that UC-MSC treatment combined with PE treatment had good safety but cannot significantly improve the short-term prognosis of patients with HBV-ACLF as compared with the single treatment. The long-term efficacy should be further evaluated. Our study is helpful for a better evaluation of the therapeutic efficacy of UC-MSCs combined with PE treatment for HBV-ACLF.

## Figures and Tables

**Table 1:** Patient's characteristics and baseline clinical features.

Parameters	Control (*n* = 30)	UC-MSC (*n* = 30)	PE (*n* = 30)	PE + UC-MSC (*n* = 20)	*P*
Sex					
Male	28 (93.33)	29 (96.67)	27 (90.00)	20 (100.00)	0.308
Female	2 (6.67)	1 (3.33)	3 (10.00)	0 (0.00)	
Age, year	44.97 ± 11.83	40.67 ± 9.89	40.87 ± 12.17	42.00 ± 6.55	0.407
Complications	19 (63.33)	16 (53.33)	21 (70.00)	11 (55.00)	0.542
Peritonitis	15 (50.00)	12 (40.00)	14 (46.67)	9 (45.00)	0.889
Pneumonia	1 (3.33)	6 (20.00)	2 (6.67)	3 (15.00)	0.144
Enteritis	2 (6.67)	3 (10.00)	3 (10.00)	2 (10.00)	0.958
Gastrointestinal bleeding	3 (10.00)	0 (0.00)	0 (0.00)	0 (0.00)	0.046
Hepatic encephalopathy	11 (36.67)	4 (13.33)	8 (26.67)	5 (25.00)	0.210
Hepatorenal syndrome	2 (6.67)	0 (0.00)	0 (0.00)	2 (10.00)	0.083
WBC, 10^9^/L	7.75 ± 3.20	5.87 ± 2.22	7.34 ± 3.13	7.88 ± 3.46	0.075
N%	67.94 ± 11.83	59.64 ± 12.21	61.21 ± 12.80	64.07 ± 10.12	0.042
RBC, 10^12^/L	3.55 ± 0.59	3.35 ± 0.70	3.62 ± 0.97	3.82 ± 0.74	0.268
Hemoglobin, g/L	113.47 ± 18.28	107.63 ± 20.34	113.63 ± 23.80	113.90 ± 18.65	0.614
Platelet, 10^9^/L	109.33 ± 66.76	100.53 ± 52.89	90.93 ± 41.98	122.80 ± 97.38	0.306
AST, U/L	260.10 ± 236.92	245.10 ± 385.06	205.13 ± 213.37	199.65 ± 188.63	0.139
ALT, U/L	373.50 ± 492.01	289.30 ± 594.25	234.57 ± 238.56	168.45 ± 149.75	0.067
Albumin, g/L	32.72 ± 3.76	34.57 ± 4.24	35.44 ± 3.79	35.60 ± 4.70	0.023
Cholinesterase, U/L	3548.40 ± 1786.64	3684.93 ± 1365.60	4068.77 ± 1070.67	4075.80 ± 1136.24	0.245
TBIL, *μ*mol/L	468.44 ± 139.43	455.78 ± 117.61	501.81 ± 135.53	542.86 ± 149.65	0.138
Creatinine, *μ*mol/L	85.04 ± 44.69	66.07 ± 18.62	76.65 ± 24.80	79.37 ± 36.90	0.096
Prothrombin time, sec.	28.99 ± 8.49	29.53 ± 6.72	32.33 ± 8.00	27.42 ± 4.32	0.097
Prothrombin activity, %	30.13 ± 7.26	27.57 ± 6.95	26.27 ± 7.48	31.40 ± 8.86	0.096
INR	2.82 ± 1.17	2.80 ± 0.83	3.22 ± 1.06	2.59 ± 0.54	0.128
MELD score	28.73 ± 4.91	26.73 ± 4.17	29.83 ± 4.93	28.10 ± 4.67	0.092
Treatment-related complications					
Fever					0.528
1 episode	—	8 (26.67)	—	5 (25.00)	
2 episodes	—	1 (3.33)	—	1 (5.00)	
3 episodes	—	2 (6.67)	—	0	
Allergic reaction					0.537
1 episode	—	—	7 (23.33)	6 (30.00)	
2 episodes	—	—	1 (3.33)	0	
Bleeding at the catheter insertion site	—	—	4 (13.33)	1 (5.00)	0.636

UC-MSC, umbilical cord-derived mesenchymal stem cells; PE, plasma exchange; WBC, white blood cells; RBC, red blood cells; AST, aspartate aminotransferase; ALT, alanine transaminase; TBIL, total bilirubin; INR, international normalized ratio; MELD, model for end-stage liver disease.

**Table 2:** Unfavorable outcome and survival rate.

Parameters	Control	UC-MSC	PE	PE + UC-MSC	All	*P*
	(*n* = 30)	(*n* = 30)	(*n* = 30)	(*n* = 20)	(*n* = 110)	
Unfavorable outcome						
30 days	12 (40.00)	5 (16.67)	7 (23.33)	2 (10.00)	26 (23.64)	0.063
60 days	16 (53.33)	16 (53.33)	12 (40.00)	5 (25.00)	49 (44.55)	0.145
90 days	16 (53.33)	18 (60.00)	16 (53.33)	7 (35.00)	57 (51.82)	0.368
180 days	18 (60.00)	19 (63.33)	17 (56.67)	9 (45.00)	63 (57.27)	0.622
360 days	18 (60.00)	19 (63.33)	18 (60.00)	10 (50.00)	65 (59.09)	0.821
Survival rate						
30 days	19 (63.33)	26 (86.67)	24 (80.00)	18 (90.00)	87 (79.09)	0.079
60 days	15 (50.00)	16 (53.33)	20 (66.67)	15 (75.00)	66 (60.00)	0.228
90 days	15 (50.00)	15 (50.00)	17 (56.67)	13 (65.00)	60 (54.55)	0.693
180 days	13 (43.33)	14 (46.67)	17 (56.67)	11 (55.00)	55 (50.00)	0.705
360 days	13 (43.33)	14 (46.67)	16 (53.33)	10 (50.00)	53 (48.18)	0.883

UC-MSC, umbilical cord-derived mesenchymal stem cells; PE, plasma exchange.

**Table 3:** Independent variables associated with unfavorable outcomes with logistic regression after modeling and adjusting covariates by GEE models.

	Univariate		Multivariate	
Parameters	OR (95% CI)	*P* value	OR (95% CI)	*P* value
UC-MSC treated				
No	ref	—	ref	—
Yes	0.73 (0.43-1.22)	0.229^1^	0.88 (0.49-1.56)	0.653
PE treated				
No	ref	—	ref	—
Yes	0.82 (0.49-1.38)	0.463^1^	0.64 (0.36-1.13)	0.124
Age, year	1.03 (1.00-1.06)	0.050	1.02 (0.99-1.06)	0.179
Sex				
Male	ref	—		
Female	0.86 (0.22-3.42)	0.828		
Complications				
No	ref	—	ref	—
Yes	2.18 (1.25-3.81)	0.006	1.09 (0.59-2.02)	0.776
WBC, 10^9^/L	1.16 (1.07-1.27)	<0.001	1.03 (0.89-1.19)	0.663
N%	1.05 (1.02-1.08)	<0.001	1.02 (0.99-1.06)	0.200
RBC, 10^12^/L	1.02 (0.99-1.06)	0.225		
Hemoglobin, g/L	0.98 (0.97-0.99)	<0.001	0.98 (0.97-1.00)	0.035
Platelet, 10^9^/L	1.00 (0.99-1.00)	0.279		
AST, U/L	1.00 (1.00-1.00)	0.033	1.00 (1.00-1.00)	0.097
ALT, U/L	1.00 (1.00-1.00)	0.219		
Albumin, g/L	1.00 (1.00-1.01)	0.173		
Cholinesterase, U/L	1.00 (1.00-1.00)	0.367	
TBIL, *μ*mol/L	1.00 (1.00-1.01)	<0.001^2^		
Creatinine, *μ*mol/L	1.01 (1.01-1.02)	0.001^2^		
Prothrombin time, sec.	1.06 (0.97-1.16)	0.172		
Prothrombin activity, %	0.88 (0.84-0.91)	<0.001	0.93 (0.87-0.98)	0.010
INR	2.24 (1.49-3.38)	<0.001^2^		
MELD score	1.23 (1.16-1.31)	<0.001	1.12 (1.03-1.22)	0.008

^1^Patients being treated with UC-MSC or/and PE would be entered into a multivariate model even if it was not significant in univariate results. ^2^TBIL, creatinine, and INR would not be included in the multivariate model because the MELD score was calculated by these three indexes to prevent multicollinearity. UC-MSC, umbilical cord-derived mesenchymal stem cells; PE, plasma exchange; WBC, white blood cells; RBC, red blood cells; AST, aspartate aminotransferase; ALT, alanine transaminase; TBIL, total bilirubin; INR, international normalized ratio; MELD, model for end-stage liver disease.

## Data Availability

The data used to support the findings of this study are included in the article.

## Abbreviations

ACLF: Acute-on-chronic liver failure
HBV-ACLF: Hepatitis B virus-related ACLF
ALSs: Artificial liver support systems
PE: Plasma exchange
MSCs: Mesenchymal stem cells
UC-MSCs: Umbilical cord-derived MSCs
HBsAg: Hepatitis B virus surface antigen
DMEM: Dulbecco's modified Eagle's medium
FFP: Fresh frozen plasma
GEE: Generalized estimating equation
AST: Aspartate aminotransferase
OR: Odds ratio.

## Supplementary Materials

Supplementary 1 Supplementary Table S1: change of biochemical markers across time in the control group (*n* = 30).

Supplementary 2 Supplementary Table S2: change of biochemical markers across time in the UC-MSC-treated group (*n* = 30).

Supplementary 3 Supplementary Table S3: change of biochemical markers across time in the PE-treated group (*n* = 30).

Supplementary 4 Supplementary Table S4: change of biochemical markers across time in the PE + UC-MSC-treated group (*n* = 20).

Supplementary 5 Supplementary Table S5: change of biochemical markers pre- and post-PE treatment in the PE-treated group (*n* = 30).

Supplementary 6 Supplementary Table S6: change of biochemical markers pre- and post-PE treatment in the PE + UC-MSC-treated group (*n* = 20).
