# Supplementary material for: Combination Treatments of Plasma Exchange and Umbilical Cord-Derived Mesenchymal Stem Cell Transplantation for Patients with Hepatitis B Virus-Related Acute-on-Chronic Liver Failure: A Clinical Trial in China
Source: Stem Cells Int. 2019 Feb 4;2019:4130757. doi: 10.1155/2019/4130757 (PMC6378797; doi:10.1155/2019/4130757)
Supplement: Supplementary 1 — Supplementary Table S1: change of biochemical markers across time in the control group (n = 30). [file 4130757.f1.docx]

**Supplementary Table S1 Change of biochemical markers across time in the control group (n=30)**

| Parameters | Baseline | 30 days | 60 days | 90 days |
| --- | --- | --- | --- | --- |
| WBC, 10^9^/L | 7.75±3.20 | 5.73±2.85 | 5.43±1.09 | 5.60±1.45 |
| N% | 67.94±11.83 | 54.67±13.12 | 52.42±14.58 | 57.20±14.16 |
| RBC, 10^12^/L | 3.55±0.59 | 3.00±0.57 | 2.89±0.64 | 2.77±0.71 |
| Hemoglobin, g/L | 113.47±18.28 | 104.06±16.71 | 102.23±20.19 | 95.14±22.09 |
| Platelet, 10^9^/L | 109.33±66.76 | 84.94±38.97 | 98.92±54.87 | 100.00±69.47 |
| AST, U/L | 260.10±236.92 | 106.50±48.56 | 89.23±51.84 | 100.00±62.37 |
| ALT, U/L | 373.50±492.01 | 54.94±21.56 | 39.62±26.95 | 41.29±46.58 |
| Albumin, g/L | 32.72±3.76 | 37.80±2.60 | 38.48±4.28 | 36.63±5.38 |
| Cholinesterase, U/L | 3548.40±1786.64 | 3476.33±1132.61 | 3807.73±1175.79 | 3072.83±1079.27 |
| TBIL, μmol/L | 468.44±139.43 | 368.61±222.04 | 311.05±202.77 | 236.04±119.66 |
| Creatinine, μmol/L | 85.04±44.69 | 93.73±51.20 | 72.29±17.87 | 92.04±55.97 |
| Prothrombin time, sec. | 28.99±8.49 | 27.22±8.31 | 43.84±57.76 | 27.54±10.80 |
| Prothrombin activity, % | 30.13±7.26 | 36.28±17.31 | 37.25±19.39 | 38.57±21.89 |
| INR | 2.82±1.17 | 2.63±1.07 | 2.61±1.17 | 2.68±1.44 |
| MELD score | 28.73±4.91 | 26.67±8.76 | 24.17±7.35 | 24.14±9.30 |

WBC, white blood cells; RBC, red blood cells; AST, aspartate aminotransferase; ALT, alanine transaminase; TBIL; total bilirubin; INR, international normalized ratio; MELD, model for end-stage liver disease.
