# Supplementary material for: Combination Treatments of Plasma Exchange and Umbilical Cord-Derived Mesenchymal Stem Cell Transplantation for Patients with Hepatitis B Virus-Related Acute-on-Chronic Liver Failure: A Clinical Trial in China
Source: Stem Cells Int. 2019 Feb 4;2019:4130757. doi: 10.1155/2019/4130757 (PMC6378797; doi:10.1155/2019/4130757)
Supplement: Supplementary 2 — Supplementary Table S2: change of biochemical markers across time in the UC-MSC-treated group (n = 30). [file 4130757.f2.docx]

**Supplementary Table S2 Change of biochemical markers across time in UC-MSC treated group (n=30)**

| Parameters | Baseline | 30 days | 60 days | 90 days |
| --- | --- | --- | --- | --- |
| WBC, 10^9^/L | 5.87±2.22 | 5.25±2.54 | 4.49±1.99 | 4.97±1.80 |
| N% | 59.64±12.21 | 60.39±11.26 | 52.52±12.41 | 50.70±12.82 |
| RBC, 10^12^/L | 3.35±0.70 | 2.75±0.55 | 2.74±0.49 | 2.99±0.67 |
| Hemoglobin, g/L | 107.63±20.34 | 94.23±15.95 | 94.64±18.30 | 99.22±18.56 |
| Platelet, 10^9^/L | 100.53±52.89 | 90.70±53.76 | 103.71±83.66 | 75.11±37.16 |
| AST, U/L | 245.10±385.06 | 153.27±256.25 | 88.07±58.96 | 71.67±28.55 |
| ALT, U/L | 289.30±594.25 | 88.03±171.07 | 46.29±25.72 | 45.00±25.71 |
| Albumin, g/L | 34.57±4.24 | 39.29±4.03 | 38.19±4.74 | 35.69±5.97 |
| Cholinesterase, U/L | 3684.93±1365.60 | 4237.60±1679.82 | 4723.86±2045.15 | 3680.00±2263.45 |
| TBIL, μmol/L | 455.78±117.61 | 460.31±164.23 | 314.23±214.56 | 240.31±256.53 |
| Creatinine, μmol/L | 66.07±18.62 | 69.62±20.75 | 62.84±20.35 | 72.67±18.77 |
| Prothrombin time, sec. | 29.53±6.72 | 30.63±11.51 | 25.35±5.95 | 24.18±6.73 |
| Prothrombin activity, % | 27.57±6.95 | 29.53±14.80 | 34.93±15.63 | 37.11±12.77 |
| INR | 2.80±0.83 | 3.01±1.60 | 2.25±0.59 | 2.10±0.57 |
| MELD score | 26.73±4.17 | 27.10±7.17 | 21.43±7.30 | 20.78±6.67 |

WBC, white blood cells; RBC, red blood cells; AST, aspartate aminotransferase; ALT, alanine transaminase; TBIL; total bilirubin; INR, international normalized ratio; MELD, model for end-stage liver disease.
