# Supplementary material for: Combination Treatments of Plasma Exchange and Umbilical Cord-Derived Mesenchymal Stem Cell Transplantation for Patients with Hepatitis B Virus-Related Acute-on-Chronic Liver Failure: A Clinical Trial in China
Source: Stem Cells Int. 2019 Feb 4;2019:4130757. doi: 10.1155/2019/4130757 (PMC6378797; doi:10.1155/2019/4130757)
Supplement: Supplementary 3 — Supplementary Table S3: change of biochemical markers across time in the PE-treated group (n = 30). [file 4130757.f3.docx]

**Supplementary Table S3 Change of biochemical markers across time in PE treated group (n=30)**

| Parameters | Baseline | 30 days | 60 days | 90 days |
| --- | --- | --- | --- | --- |
| WBC, 10^9^/L | 7.34±3.13 | 5.67±3.46 | 5.95±2.97 | 3.79±0.93 |
| N% | 61.21±12.80 | 53.50±13.17 | 51.67±12.30 | 53.50±6.58 |
| RBC, 10^12^/L | 3.62±0.97 | 3.59±3.92 | 2.78±1.04 | 3.09±1.55 |
| Hemoglobin, g/L | 113.63±23.80 | 95.84±17.88 | 91.33±27.91 | 95.25±28.19 |
| Platelet, 10^9^/L | 90.93±41.98 | 79.28±32.59 | 91.25±53.73 | 76.75±39.66 |
| AST, U/L | 205.13±213.37 | 82.12±56.13 | 68.33±49.27 | 39.50±5.69 |
| ALT, U/L | 234.57±238.56 | 46.32±39.74 | 39.92±30.45 | 25.25±10.63 |
| Albumin, g/L | 35.44±3.79 | 38.42±2.92 | 36.83±4.94 | 39.60±3.60 |
| Cholinesterase, U/L | 4068.77±1070.67 | 4739.17±1448.12 | 4381.00±1573.97 | 5342.75±1527.73 |
| TBIL, μmol/L | 501.81±135.53 | 374.11±255.14 | 368.45±319.90 | 247.03±198.14 |
| Creatinine, μmol/L | 76.65±24.80 | 85.45±75.73 | 66.78±16.26 | 73.85±19.69 |
| Prothrombin time, sec. | 32.33±8.00 | 32.04±15.61 | 31.85±13.77 | 29.28±11.57 |
| Prothrombin activity, % | 26.27±7.48 | 31.76±15.32 | 34.25±20.79 | 36.25±22.74 |
| INR | 3.22±1.06 | 3.27±2.19 | 3.20±1.78 | 2.78±1.28 |
| MELD score | 29.83±4.93 | 26.96±10.83 | 24.58±9.58 | 23.00±10.36 |

WBC, white blood cells; RBC, red blood cells; AST, aspartate aminotransferase; ALT, alanine transaminase; TBIL; total bilirubin; INR, international normalized ratio; MELD, model for end-stage liver disease.
