# Supplementary material for: Combination Treatments of Plasma Exchange and Umbilical Cord-Derived Mesenchymal Stem Cell Transplantation for Patients with Hepatitis B Virus-Related Acute-on-Chronic Liver Failure: A Clinical Trial in China
Source: Stem Cells Int. 2019 Feb 4;2019:4130757. doi: 10.1155/2019/4130757 (PMC6378797; doi:10.1155/2019/4130757)
Supplement: Supplementary 4 — Supplementary Table S4: change of biochemical markers across time in the PE + UC-MSC-treated group (n = 20). [file 4130757.f4.docx]

**Supplementary Table S4 Change of biochemical markers across time in PE+UC-MSC treated group (n=20)**

| Parameters | Baseline | 30 days | 60 days | 90 days |
| --- | --- | --- | --- | --- |
| WBC, 10^9^/L | 7.88±3.46 | 6.38±2.74 | 6.26±3.35 | 4.14±1.02 |
| N% | 64.07±10.12 | 60.79±11.71 | 51.03±18.55 | 47.97±13.74 |
| RBC, 10^12^/L | 3.82±0.74 | 2.92±0.61 | 8.42±21.18 | 2.60±0.21 |
| Hemoglobin, g/L | 113.90±18.65 | 96.58±17.22 | 93.50±14.50 | 87.83±15.26 |
| Platelet, 10^9^/L | 122.80±97.38 | 98.16±80.33 | 83.86±28.42 | 73.33±22.94 |
| AST, U/L | 199.65±188.63 | 105.26±46.48 | 98.50±47.03 | 68.83±25.25 |
| ALT, U/L | 168.45±149.75 | 68.26±37.40 | 50.86±21.76 | 48.00±18.54 |
| Albumin, g/L | 35.60±4.70 | 59.82±87.53 | 82.24±173.19 | 35.45±4.75 |
| Cholinesterase, U/L | 4075.80±1136.24 | 5114.47±1579.55 | 3992.43±1440.23 | 2907.50±1232.84 |
| TBIL, μmol/L | 542.86±149.65 | 458.33±281.71 | 374.16±289.44 | 226.25±271.47 |
| Creatinine, μmol/L | 79.37±36.90 | 72.89±21.27 | 90.99±96.16 | 60.72±15.56 |
| Prothrombin time, sec. | 27.42±4.32 | 29.30±8.21 | 32.51±12.18 | 27.07±7.80 |
| Prothrombin activity, % | 31.40±8.86 | 31.00±13.39 | 27.71±10.41 | 34.17±12.22 |
| INR | 2.59±0.54 | 2.83±0.97 | 3.24±1.66 | 2.54±0.95 |
| MELD score | 28.10±4.67 | 26.84±6.96 | 26.93±11.19 | 20.00±8.76 |

WBC, white blood cells; RBC, red blood cells; AST, aspartate aminotransferase; ALT, alanine transaminase; TBIL; total bilirubin; INR, international normalized ratio; MELD, model for end-stage liver disease.
