# Supplementary material for: Combination Treatments of Plasma Exchange and Umbilical Cord-Derived Mesenchymal Stem Cell Transplantation for Patients with Hepatitis B Virus-Related Acute-on-Chronic Liver Failure: A Clinical Trial in China
Source: Stem Cells Int. 2019 Feb 4;2019:4130757. doi: 10.1155/2019/4130757 (PMC6378797; doi:10.1155/2019/4130757)
Supplement: Supplementary 5 — Supplementary Table S5: change of biochemical markers pre- and post-PE treatment in the PE-treated group (n = 30). [file 4130757.f5.docx]

**Supplementary Table S5 Change of biochemical markers pre- and post- PE treatment in PE treated group (n=30)**

|  | First | | |  | Second | | |  | Third | | |
| --- | --- | --- | --- | --- | --- | --- | --- | --- | --- | --- | --- |
| Parameters | Pre-treatment | Post-treatment | P |  | Pre-treatment | Post-treatment | P |  | Pre-treatment | Post-treatment | P |
| WBC, 10^9^/L | 6.03±2.67 | 5.24±1.95 | 0.021 |  | 6.36±2.41 | 5.27±2.08 | <0.001 |  | 6.10±3.15 | 5.50±2.84 | 0.025 |
| N% | 63.04±15.03 | 70.80±15.49 | <0.001 |  | 61.56±13.36 | 69.93±13.97 | <0.001 |  | 59.73±7.61 | 69.29±8.90 | <0.001 |
| RBC, 10^12^/L | 3.26±0.94 | 3.06±0.83 | <0.001 |  | 3.17±0.91 | 2.92±0.78 | <0.001 |  | 3.07±0.85 | 2.92±0.80 | <0.001 |
| Hemoglobin, g/L | 103.53±23.72 | 97.20±20.97 | <0.001 |  | 101.80±21.82 | 94.03±17.89 | <0.001 |  | 100.08±18.28 | 95.46±17.88 | <0.001 |
| Platelet, 10^9^/L | 88.27±41.41 | 77.57±44.55 | 0.001 |  | 78.40±44.30 | 67.07±39.74 | <0.001 |  | 73.63±34.59 | 63.92±35.70 | <0.001 |
| AST, U/L | 180.93±238.99 | 96.77±102.44 | <0.001 |  | 124.43±142.82 | 74.17±70.99 | <0.001 |  | 102.29±53.05 | 83.29±104.12 | <0.001 |
| ALT, U/L | 155.57±184.69 | 83.43±84.22 | <0.001 |  | 92.47±100.68 | 55.83±51.07 | <0.001 |  | 72.83±68.19 | 45.50±36.20 | <0.001 |
| Albumin, g/L | 34.78±4.02 | 33.79±2.94 | 0.049 |  | 34.48±4.14 | 34.20±2.10 | 0.758 |  | 36.25±4.34 | 34.99±2.77 | 0.067 |
| Cholinesterase, U/L | 3886.63±1139.95 | 5112.37±642.38 | <0.001 |  | 4632.17±1201.04 | 5566.70±628.89 | <0.001 |  | 4907.17±826.12 | 5634.21±613.09 | <0.001 |
| TBIL, μmol/L | 495.54±127.59 | 313.90±98.09 | <0.001 |  | 442.28±129.19 | 275.98±86.50 | <0.001 |  | 398.22±146.58 | 240.15±97.29 | <0.001 |
| Creatinine, μmol/L | 73.77±20.28 | 76.91±23.26 | 0.056 |  | 74.96±26.09 | 78.09±26.96 | 0.033 |  | 71.63±18.99 | 74.09±15.73 | 0.087 |
| Prothrombin time, sec. | 33.56±10.57 | 18.22±1.18 | <0.001 |  | 31.44±9.37 | 17.96±1.16 | <0.001 |  | 28.97±7.39 | 17.63±1.41 | <0.001 |
| Prothrombin activity, % | 25.77±7.82 | 55.47±5.84 | <0.001 |  | 28.17±9.33 | 56.73±6.42 | <0.001 |  | 30.92±10.14 | 58.75±7.83 | <0.001 |
| INR | 3.39±1.41 | 1.52±0.13 | <0.001 |  | 3.11±1.21 | 1.49±0.13 | <0.001 |  | 2.80±0.94 | 1.45±0.15 | <0.001 |
| MELD score | 30.03±5.31 | 20.23±3.40 | <0.001 |  | 28.57±5.52 | 19.53±3.54 | <0.001 |  | 26.67±5.35 | 18.50±3.05 | <0.001 |

WBC, white blood cells; RBC, red blood cells; AST, aspartate aminotransferase; ALT, alanine transaminase; TBIL; total bilirubin; INR, international normalized ratio; MELD, model for end-stage liver disease.
