# Supplementary material for: Combination Treatments of Plasma Exchange and Umbilical Cord-Derived Mesenchymal Stem Cell Transplantation for Patients with Hepatitis B Virus-Related Acute-on-Chronic Liver Failure: A Clinical Trial in China
Source: Stem Cells Int. 2019 Feb 4;2019:4130757. doi: 10.1155/2019/4130757 (PMC6378797; doi:10.1155/2019/4130757)
Supplement: Supplementary 6 — Supplementary Table S6: change of biochemical markers pre- and post-PE treatment in the PE + UC-MSC-treated group (n = 20). [file 4130757.f6.docx]

**Supplementary Table S6 Change of biochemical markers pre- and post- PE treatment in PE+UC-MSC treated group (n=20)**

|  | First | | |  | Second | | |  | Third | | |
| --- | --- | --- | --- | --- | --- | --- | --- | --- | --- | --- | --- |
| Parameters | Pre-treatment | Post-treatment | P |  | Pre-treatment | Post-treatment | P |  | Pre-treatment | Post-treatment | P |
| WBC, 10^9^/L | 7.24±3.13 | 6.23±2.92 | 0.002 |  | 6.39±2.92 | 6.27±3.02 | 0.185 |  | 6.40±2.75 | 6.11±2.33 | 0.339 |
| N% | 67.38±9.74 | 76.03±7.75 | <0.001 |  | 68.50±8.45 | 75.07±8.27 | <0.001 |  | 69.29±6.70 | 74.84±8.15 | <0.001 |
| RBC, 10^12^/L | 3.39±0.74 | 3.14±0.66 | <0.001 |  | 3.25±0.69 | 3.13±0.75 | 0.028 |  | 3.12±0.73 | 2.97±0.77 | 0.004 |
| Hemoglobin, g/L | 103.10±17.60 | 97.60±18.32 | 0.001 |  | 99.75±16.82 | 96.95±18.84 | 0.076 |  | 96.94±18.41 | 92.82±20.12 | 0.012 |
| Platelet, 10^9^/L | 110.65±75.23 | 100.20±76.45 | 0.005 |  | 94.45±64.91 | 80.80±59.70 | <0.001 |  | 87.35±51.97 | 77.18±49.06 | <0.001 |
| AST, U/L | 165.05±146.60 | 96.55±65.36 | <0.001 |  | 128.15±91.99 | 85.45±72.63 | <0.001 |  | 132.24±85.70 | 88.94±80.25 | <0.001 |
| ALT, U/L | 125.15±104.85 | 74.25±49.85 | <0.001 |  | 84.15±60.09 | 61.05±57.12 | <0.001 |  | 76.47±55.91 | 54.76±57.07 | <0.001 |
| Albumin, g/L | 34.98±4.39 | 33.91±3.65 | 0.057 |  | 35.15±3.69 | 34.33±3.06 | 0.243 |  | 35.86±4.70 | 34.77±3.53 | 0.083 |
| Cholinesterase, U/L | 3649.22±1190.48 | 4927.25±575.48 | <0.001 |  | 4651.20±861.86 | 5312.55±1023.93 | 0.007 |  | 4883.59±1156.41 | 5486.94±1316.90 | <0.001 |
| TBIL, μmol/L | 530.12±183.66 | 335.46±121.00 | <0.001 |  | 468.30±159.11 | 283.80±102.36 | <0.001 |  | 434.50±161.60 | 267.21±101.41 | <0.001 |
| Creatinine, μmol/L | 84.46±48.31 | 84.80±42.58 | 0.266 |  | 80.10±34.26 | 79.54±34.57 | 0.679 |  | 73.35±22.49 | 72.16±20.12 | 0.501 |
| Prothrombin time, sec. | 28.39±4.96 | 18.15±1.43 | <0.001 |  | 27.17±5.06 | 18.17±1.65 | <0.001 |  | 27.01±6.00 | 18.06±1.86 | <0.001 |
| Prothrombin activity, % | 30.35±10.79 | 55.70±6.97 | <0.001 |  | 32.30±10.72 | 55.85±7.62 | <0.001 |  | 33.00±11.73 | 56.47±9.08 | <0.001 |
| INR | 2.72±0.62 | 1.51±0.16 | <0.001 |  | 2.56±0.62 | 1.51±0.17 | <0.001 |  | 2.57±0.77 | 1.51±0.20 | <0.001 |
| MELD score | 28.70±5.20 | 20.80±4.71 | <0.001 |  | 27.30±5.25 | 19.60±4.36 | <0.001 |  | 26.41±4.80 | 18.82±3.84 | <0.001 |

WBC, white blood cells; RBC, red blood cells; AST, aspartate aminotransferase; ALT, alanine transaminase; TBIL; total bilirubin; INR, international normalized ratio; MELD, model for end-stage liver disease.
